# Supplementary material for: Unraveling Spurious Properties of Interaction Networks with Tailored Random Networks
Source: PLoS One. 2011 Aug 5;6(8):e22826. doi: 10.1371/journal.pone.0022826 (PMC3151270; doi:10.1371/journal.pone.0022826)
Supplement: Appendix S1 — Mathematical proofs. (PDF) [file pone.0022826.s001.pdf]

Text Supplementary 1 – Unraveling Spurious Properties of Interaction Networks with Tailored Random Networks

Stephan Bialonski[1,2,3,*], Martin Wendler[4], Klaus Lehnertz[1,2,3]

1 Department of Epileptology, University of Bonn, Bonn, Germany

2 Helmholtz Institute for Radiation and Nuclear Physics, University of Bonn, Bonn, Germany

3 Interdisciplinary Center for Complex Systems, University of Bonn, Bonn, Germany

4 Fakultät für Mathematik, Ruhr-Universität Bochum, Bochum, Germany

∗ E-mail: bialonski@gmx.net

Lemma 1

For every $i, j \in \{1, \ldots, N\}$ with $i \neq j$, we have the following limit of the probability distribution of the empirical correlation:

$$P\left(\sqrt{\frac{T}{g(M)}}\mathrm{corr}(x_{i,M,T}, x_{j,M,T}) \leq x\right) \to \Phi(x) \quad \text{with } g(M) = \frac{2}{3}M + \frac{1}{3}\frac{1}{M} \tag{1}$$

as $T \to \infty$, where Φ denotes the cumulative distribution function of a standard normal random variable.

Proof

In order to simplify the presentation, we write $y_{i,M,T}(t) = x_{i,M,T}(t) - \frac{1}{2}$, so that $Ey_{i,M,T}(t) = 0$. First note that $y_{i,M,T}(t)$ is a M-dependent sequence, i.e. for $|s-t| > M$, $y_{i,M,T}(s)$ and $y_{i,M,T}(t)$ are independent. So we have that the covariance

$$\mathrm{Cov}\left(y_{i,M,T}(1)y_{j,M,T}(1), y_{i,M,T}(t)y_{j,M,T}(t)\right) = 0 \qquad \text{for } T > M.$$

Additionally,

$$\mathrm{Cov}\left(y_{i,M,T}(1)y_{j,M,T}(1), y_{i,M,T}(t)y_{j,M,T}(t)\right) = \mathrm{Cov}\left(y_{i,M,T}(1), y_{i,M,T}(t)\right)\mathrm{Cov}\left(y_{j,M,T}(1), y_{j,M,T}(t)\right) \tag{2}$$

and $\mathrm{Cov}\left(z_i(s), z_i(t)\right) = \mathrm{Var}\left(z_i(1)\right)$ if $s = t$ and otherwise $\mathrm{Cov}\left(z_i(s), z_i(t)\right) = 0$. For $1 \leq t \leq M$, we obtain by the definition of the moving average and the independence of the underlying process $z_j(t)$, $t \in \mathbb{N}$ that

$$\mathrm{Cov}\left(y_{i,M,T}(1)y_{j,M,T}(1), y_{i,M,T}(t)y_{j,M,T}(t)\right) = \frac{1}{M^4}\left(\sum_{s=1}^{M-(t-1)} \mathrm{Var}\left(z_j(s)\right)\right)^2 \tag{3}$$

$$= \frac{1}{M^4}(M-(t-1))^2 \mathrm{Var}^2\left(z_i(1)\right). \tag{4}$$

By the central limit theorem for M-dependent random variables, see reference [1],

$$\frac{1}{\sqrt{\mathrm{Var}\left(\frac{1}{T}\sum_{t=1}^{T} y_{i,M,T}(t)y_{j,M,T}(t)\right)}}\frac{1}{T}\sum_{t=1}^{T} y_{i,M,T}(t)y_{j,M,T}(t) \tag{5}$$

converges in distribution to a standard normal random varibale as $T \to \infty$. Furthermore, we have the following convergence for the variance as $T \to \infty$:

$$\begin{aligned} &T\mathrm{Var}\left(\frac{1}{T}\sum_{t=1}^{T} y_{i,M,T}(t)y_{j,M,T}(t)\right) \\ &\quad \to \mathrm{Var}(y_{i,M,T}(1)y_{j,M,T}(1)) + 2\sum_{t=2}^{M} \mathrm{Cov}\left(y_{i,M,T}(1)y_{j,M,T}(1), y_{i,M,T}(t)y_{j,M,T}(t)\right) \\ &\quad = \left(\frac{1}{M^2} + \frac{2}{M^4}\sum_{t=2}^{M}(M-(t-1))^2\right)\mathrm{Var}^2\left(z_i(1)\right) = \frac{g(M)}{M^2}\mathrm{Var}^2\left(z_i(1)\right). \end{aligned} \tag{6}$$

The last equality follows easily by $\sum_{i=1}^{n} i^2 = \frac{n(n+1)(2n+1)}{6}$. With the same central limit theorem, $\frac{1}{\sqrt{T}}\sum_{t=1}^{T} y_{i,M,T}(t)$ converges to a normal limit, so $\frac{1}{T^{\frac{3}{4}}}\sum_{t=1}^{T} y_{i,M,T}(t) \to 0$ in probability and consequently

$$\sqrt{T}\left(\frac{1}{T}\sum_{t=1}^{T} y_{i,M,T}(t)\right)\left(\frac{1}{T}\sum_{t=1}^{T} y_{j,M,T}(t)\right) = \left(\frac{1}{T^{\frac{3}{4}}}\sum_{t=1}^{T} y_{i,M,T}(t)\right)\left(\frac{1}{T^{\frac{3}{4}}}\sum_{t=1}^{T} y_{j,M,T}(t)\right) \to 0 \tag{7}$$

in probability as $T \to \infty$. By similar arguments, we have that $\frac{1}{T}\sum_{t=1}^{T} y_{i,M,T}^2(t) \to \mathrm{Var}(y_{i,M,T}(1)) = \frac{1}{M}\mathrm{Var}\left(z_i(1)\right)$ and $\frac{1}{T}\sum_{t=1}^{T} y_{i,M,T}(t) \to 0$, so we get

$$\frac{1}{T}\sum_{t=1}^{T}(y_{i,M,T}(t) - \bar{y}_{i,M,T})^2 = \frac{1}{T}\sum_{t=1}^{T} y_{i,M,T}^2(t) - \left(\frac{1}{T}\sum_{t=1}^{T} y_{i,M,T}(t)\right)^2$$
$$\to \mathrm{Var}(y_{i,M,T}(1)) = \frac{1}{M}\mathrm{Var}\left(z_i(1)\right). \quad (8)$$

By Slutsky's theorem [2] and with (5), (6), (7), and (8), we finally obtain that

$$\sqrt{\frac{T}{g(M)}}\mathrm{corr}(x_{i,M,T}, x_{j,M,T})$$
$$= \frac{\sqrt{T}\frac{1}{T}\sum_{t=1}^{T} y_{i,M,T}(t)y_{j,M,T}(t) - \sqrt{T}\left(\frac{1}{T}\sum_{t=1}^{T} y_{i,M,T}(t)\right)\left(\frac{1}{T}\sum_{t=1}^{T} y_{j,M,T}(t)\right)}{\sqrt{g(M)\frac{1}{T}\sum_{t=1}^{T}(y_{i,M,T}(t) - \bar{y}_{i,M,T})^2\frac{1}{T}\sum_{t=1}^{T}(y_{j,M,T}(t) - \bar{y}_{j,M,T})^2}} \quad (9)$$

converges in distribution to a standard normal random variable as $T \to \infty$. This completes the proof.

Lemma 2

For $T \to \infty$, $R \to \infty$

$$\hat{\epsilon}\left(\frac{\theta}{\sqrt{T_{\mathrm{eff}}(M)}}, M, T\right) \to 2\Phi(-\theta) \quad (10)$$

in probability with $T_{\mathrm{eff}}(M) = \frac{T}{g(M)}$.

Proof

With Lemma 1, we have that

$$E\left[H_{ij,M,T}^{(r)}\left(\frac{\theta}{\sqrt{T_{\text{eff}}(M)}}\right)\right] = P\left(\rho_{ij,M,T} > \frac{\theta}{\sqrt{T_{\text{eff}}(M)}}\right)$$
$$= P\left(\text{corr}(x_{i,M,T}, x_{j,M,T}) > \frac{\theta}{\sqrt{T_{\text{eff}}(M)}}\right) + P\left(\text{corr}(x_{i,M,T}, x_{j,M,T}) < \frac{-\theta}{\sqrt{T_{\text{eff}}(M)}}\right)$$
$$= P\left(\sqrt{\frac{T}{g(M)}}\rho_{ij,M,T} > \theta\right) + P\left(\sqrt{\frac{T}{g(M)}}\rho_{ij,M,T} < -\theta\right) \to 2\Phi(-\theta)$$

as $T \to \infty$. Furthermore, $H_{ij,M,T}^{(r)}$ is bounded by 0 and 1, so $\text{Var}\left(H_{ij,M,T}^{(r)}\right) \leq \frac{1}{4}$. By the independence of the R random networks

$$\text{Var}\left(\hat{e}\left(\frac{\theta}{\sqrt{T_{\text{eff}}(M)}}, M, T\right)\right) = \frac{1}{R^2}\sum_{r=1}^{R}\text{Var}\left(H_{ij,M,T}^{(r)}\left(\frac{\theta}{\sqrt{T_{\text{eff}}(M)}}\right)\right) \leq \frac{1}{4R} \to 0 \quad (11)$$

as $R \to \infty$. The lemma follows with the Chebyshev inequality.

References

1. Hoeffding W, Robbins H (1948) The central limit theorem for dependent random variables. Duke Math J 15: 773–780.

2. Slutsky E (1925) Über stochastische Asymptoten und Grenzwerte (in german). Metron 5: 3–89.
